# Perchlorate Exposure and Thyroid Function in Ammonium Perchlorate Workers in Yicheng, China

**DOI:** 10.3390/ijerph110504926

**Published:** 2014-05-07

**Authors:** Hongxia Chen, Libing Wu, Xia Wang, Qin Liu, Miaohong Ding, Kailiang Peng, Zhongji Meng

**Affiliations:** 1 Institute of Biomedicine, Taihe Hospital, Hubei University of Medicine, Shiyan 442000, China; E-Mail: hustchenhx@163.com; 2 School of Public Health, Tongji Medical College, Huazhong University of Science and Technology, Wuhan 430030, China; E-Mails: woodside@163.com (Q.L.); dingmiaohong@163.com (M.D.); 3 Department of Nuclear Medicine, Taihe Hospital, Hubei University of Medicine, Shiyan 442000, China; E-Mail: wuerfan@sina.com; 4 Department of Maternal and Child Health Care, School of Public Health, Shandong University, Jinan 250012, China; E-Mail: wangxiaes@sdu.edu.cn

**Keywords:** ammonium perchlorate, thyroid, occupational exposure, biomarker

## Abstract

The impact of low level dust on the thyroid function of workers chronically exposed to ammonium perchlorate (AP) is uncertain and controversial. The aim of this study was to examine whether workers in China with long-term (>3 years) occupational exposure to low levels of AP dust had affected thyroid homeostasis. Mean occupational exposures to AP dust ranged from 0.43 to 1.17 mg/m^3^. Geometric means of post-shift urinary perchlorate levels were 20.5 µg/L for those exposed and 12.8 µg/L for the controls. No significant differences were found for thyroid function parameters of FT_3_, FT_4_, or log TSH or for TPO prevalence or thyroglobulin levels. Additionally, no differences in findings were observed for complete blood count (CBC), serum biochemical profile, or pulmonary function test. Median urinary iodine levels of 172 and 184 µg/L showed that the workers had sufficient iodine intake. This study found no effect on thyroid function from long term, low-level documented exposure to ammonium perchlorate. It is the first study to report both thyroid status parameters and urinary perchlorate, a biomarker of internal perchlorate exposure, in occupationally exposed workers in China.

## 1. Introduction

Ammonium perchlorate (AP) is a strong oxidant widely employed in military and civilian energy materials (propellants). Recently, it has been scrutinized as a persistent environmental pollutant [1] due to its wide use in fireworks, paints, coatings and other civilian industrial products. The presence of perchlorate in the environment is of both natural [2,3,4], and anthropogenic origins, via improper waste disposal associated with production and use. Exposure of humans to perchlorate via foodstuffs and drinking water has been widely documented [5]. Additionally, exposure can also derive from inhalation of dust containing AP, especially in workplaces [6]. The prevalence of trace levels of perchlorate in the environment probably leads to human exposure [7]. Perchlorate is well known as a potent inhibitor of iodide uptake due to a similar ionic radius as iodide [8]. Its toxicity is mainly reflected in the inhibition of the sodium iodide symporter (NIS), which results in reduced iodide uptake. Continuous inhibition can produce intracellular iodine deficiency, as that iodine is the important precursor for thyroid hormone synthesis. Long-term exposure to perchlorate may leads to insufficient production of thyroid hormones. Thyroid hormone homeostasis is usually met through upregulation of NIS via the hypothalamus-pituitary-thyroid (H-P-T) feedback mechanism [9]. Many animal studies have shown that perchlorate can inhibit thyroidal iodide and lead to a decrease of serum thyroid hormone level and increase of serum TSH level in response to iodine insufficiency [10,11,12].

Perchlorate has been used historically in pharmacologic doses to treat hyperthyroidism, and the ability of perchlorate to inhibit thyroid iodide uptake by the sodium-iodide symporter (NIS) has been tested *in vitro* [13]. However, the impacts on thyroid function of long-term low-dose exposures to perchlorate are uncertain and controversial. To our knowledge there is no convincing epidemiological evidence for adverse health effects in human beings through exposure to perchlorate [14], such that the debate now focuses on the potential toxicity of low level perchlorate exposure on the general population [15]. Furthermore, a study has pointed out that any discussion about safe levels of perchlorate must be framed in the context of recent study data concerning iodine nutrition status [1,16]. Previous research has shown that thyroid dysfunction induced by perchlorate in individuals with low iodine intake was exacerbated [17].

China was formerly an iodine-deficient country, with 40% of the World’s iodine deficient population. Therefore, the Chinese government implemented a program of distributing iodized salt in 1996, with the aim of eliminating iodine deficiency by 2010. However until now, there have been no reports on the iodine nutrition status associated with occupational exposure to perchlorate in China. China also has large quantities, and increased use of fireworks leaving many workers being exposed to perchlorate. Human studies on perchlorate include cross-sectional epidemiology studies of perchlorate workers with inhalation exposure, and many studies found no significant effects on thyroid homeostasis associated with perchlorate dust exposure [17,18]. The possible reason was that there was an adequate supply of iodine in those areas, thus compensating for the perchlorate exposure-related downregulation of iodine uptake.

The present study aimed to examine whether AP dust exposure has an impact on the thyroid homeostasis of occupationally exposed workers. The study was conducted on the workers of the ammonium perchlorate manufacturing company 525 Investment Co., Ltd. Yicheng City, Hubei, which has the second largest manufacturing operation in China. This is the first study to explore the potential biomarkers of occupational AP exposure in Chinese workers, especially those with long-term, low level of AP dust exposure.

## 2. Methods

### 2.1. Ethics Statement

The study protocol and the questionnaire were reviewed and approved by the ethics committee of Huazhong University of Science and Technology. All the participants were volunteers and submitted written informed consent before being involved in the study. 

### 2.2. Population and Design

This is a cross-sectional study carried out at an ammonium perchlorate production factory in Yicheng City, Hubei Province, during the period of 8 April to 11 April 2012. The plant is the second largest factory in the country with an annual output of 1500 tons. Subjects were enrolled after completion of a normal physical examination that including thyroid function and lung functional tests. The age and gender of the participants and the date of blood and urine collection were recorded for each sample, and information was also collected regarding as education status, self-reported cigarette smoking, history of occupational exposure, dietary habits, lifestyle, and personal medical history. The workers with continuous service for more than three years were choosing for the exposed group, whereas the workers un-exposed to AP dust in the same factory were selected as the control group. This detailed demographic information including gender, age, body mass index (BMI), working years, and smoking status is shown in Table 1. Shift work for 8 hours was carried out in the factory. Fasting venous blood and morning urine were collected and stored in polypropylene containers at −20 °C until analysis. In addition, to validate the efficiency of urinary perchlorate as an internal exposure indicator, both the urine samples of occupational workers before the shift and at the end of shift were collected and tested.

**Table 1:** Basic demographic characteristics of workers from the exposed group and control group (Mean ± SD).

Parameters	Exposed Group (n = 51)	Control Group (n = 41)	*p-*Values
Gender (male/female)	46/5	32/9	0.15
Age (year)	46.61 ± 10.34	44.05 ± 6.12	0.144
Height (cm)	162.20 ± 24.22 ^a^	163.98 ± 7.12	0.651
Weight (kg)	59.88 ± 13.47 ^a^	62.16 ± 9.54	0.363
BMI (body mass index)	21.65 ± 4.04 ^a^	23.04 ± 2.59	0.087
Working years (year)	11.49 ± 6.70	12.05 ± 9.11	0.326
Smoking (cigarettes/day)	6.22 ± 9.61	4.12 ± 8.10	0.27
Proportion of smokers (%)	39.2	41.5	0.83
Smoking by smokers(cigs/day)	15 ± 3.2	9.9 ± 1.5	1.0

Note: ^a^ missing values, n = 50.

### 2.3. Measures

Routine blood and serum biochemical profiles were measured. Morning spot urine samples were measured for perchlorate, iodine and creatinine, as well as the routine urinalysis. Additionally, thyroid function parameters were measured in duplicate, in the same assay and in random order. Routine blood and serum biochemical profiles were measured by standard methods in Dongfang Hospital, Yicheng, Hubei. Thyroid function tests were carried out in the Nuclear Medicine Research Laboratory of Taihe Hospital (Shiyan, China) with the normal values in parentheses: TSH (0.7–7.59 μU/mL), FT_3 _(3.50–8.56 pmol/L), FT_4_ (8.56–25.60 pmol/L), TT_3_ (0.9–2.2 ng/mL), TT_4_ (45–135 ng/mL), and Tg (11.41–20.25 ng/mL), respectively. The assays were conducted using radio-immunoassay (RIA) kits (Northern Biological Co. Ltd., Beijing, China) following the manufacturers’ instructions. The RIA kits used in this study contain standard reference preparations which were serially diluted for the standard curves, and the minimum sensitivity of RIAs was 0.5 fmol/mL for FT_3_, 1 fmol/mL for FT_4_, 0.2 ng/mL for TT_3_, 5 ng/mL for TT_4_, 3 ng/mL for Tg, and less than 0.8 μIU/mL for TSH, respectively. For each index evaluated, RIA kits with the same lot number and expiry date were used. Tracer (^125^I) radioactivity was measured using a γ counter (GC-1200 gamma radio-immunoassay counter, Anhui, China). Urinary creatinine and iodine tests were carried out in the Public Health Experiment Laboratories of Tongji Medical College, Huazhong University of Science and Technology (Wuhan, China). Casual urinary samples were detected for iodine concentration by a colorimetric method [19] using an automatic analyzer apparatus (Olympus, Tokyo, Japan). The results were calculated as micrograms of iodine per L urine and were expressed as the median. Creatinine adjustment was applied to exclude the impact of confounding factors such as difference in water intake. Urinary creatinine was measured by a kinetic JaffeA method [20].

### 2.4. Exposure

The exposure factor of primary interest in this study was ammonium perchlorate concentration in dust in the workplace, which was monitored consecutively for two days at the location of major production sources of dust in the factory (Table 2). A FC-2 dust sampler (Wuhan, China) was employed for long fixed-point sampling, and a BH-CQ1050 individual sampler (Wuhan, China) was used to determine the concentration of the dust for the time weighted average (PC-TWA) in the breathing zone (an appropriate height of 1.5 meters from the ground) in the major areas of dust production where the workers were exposed. Sampling condition and sampling strategy were based on the Method [21] (GBZ/T192.1-2007), which was approved by Ministry of Health of the People’s Republic of China. The method involved collection of the AP total dust in the air on to the membrane known mass (prior to being dried and weighed) for later calculating the concentration of total dust in the air by the incremental membrane and gas production (Gas flow rate multiplied by the time taken). The sampling flow was validated by the portable electronic soap film flow meter (TH-ZM8, Wuhan, China).

**Table 2:** Total dust concentrations of major workshop in the factory for producing ammonium perchlorate (Mean ± SD).

Locations	Sample Number	Total Dust Concentration(mg/m^3^) *
Blending and homogenization workshop	4	0.51 ± 0.002
Drying sifting workshop	8	1.18 ± 0.02
Weighing workshop	4	1.18 ± 0.01
Double decomposition workshop	4	0.42 ± 0.00

Notes: * these data are the area samples, and the duration of the TWA samples for a short time sampling varying from 20 to 30 min (GBZ/T192.1-2007).

### 2.5. Instrumental Analysis

The concentration of ammonium perchlorate in the urine samples was quantitatively analyzed to distinguish the exposed from the unexposed group, using the initial established method of the ultra-high performance liquid chromatography mass spectrometry/mass spectrometry (UHPLC-MS/MS) based on the previously reported method [22]. The experiment was facilitated at the Centers for Disease Control and Prevention, Hubei Province, China.

### 2.6. Statistical Analysis

The distributions of demographic variables of the two groups were compared using a chi-square test for categorical variables and a t-test for continuous variables. As both TSH levels and urinary perchlorate non-normal distributions, their analyses were carried out after logarithmic transformation. The data were analyzed with SPSS 15.0 (Chicago, IL, USA). The results were presented as mean ± SD and median (range), and two- tailed significance was set at *p* < 0.05.

## 3. Results

### 3.1. Demographic Characteristics

There were 51 workers in the exposed group and 41 among the controls. They did not differ significantly in proportion of males (90% *vs*. 78%; *p* = 0.15), age (46.6 *vs*. 44.1; *p* = 0.14), working years (11.5 *vs.* 12.1; *p* = 0.33), height in cm (162.2 *vs*. 164.0; *p* = 0.65), weight in kg (59.9 *vs*. 62.2; *p* = 0.36), or proportion of smokers (39.2% (n = 20) *vs*. 41.5% (n = 17); *p* = 0.83). The exposed workers had borderline lower mean BMI (21.7 *vs*. 23.0; *p* = 0.05). The average daily cigarette consumption of all exposed workers was slightly greater than that of all of the controls (6.22 *vs.* 4.12; *p* = 0.27), but the difference is not statistically significant. Nor was the average daily cigarette consumption of exposed workers who smoked significantly greater (15 *vs*. 9.9, *p* = 1.0) than that for the controls who smoked. The study sample included only one individual (in the control group) who reported a history of thyroid disease; this subject had been diagnosed with thyroid nodule ten years previously but was not taking thyroid medications.

### 3.2. Environmental Exposure

As shown in Table 2, a low-level dust exposure (less than 5 mg/m^3^, this standard has passed examination and approval, and is now awaiting being issued by the Ministry of Health of the People’s Republic of China) in the ammonium perchlorate manufacturing workshop was verified (max: 1.18 mg/m^3^; min: 0.42 mg/m^3^), due to the use of sealed against dust hoods and personal dust masks. The sensitivity of the measurements of the methods (GBZ/T192.1-2007) is represented with the lowest detection concentration of 0.2 mg/m^3^. 

### 3.3. Individual Exposure

The mean value of urinary perchlorate in the exposed group was obviously higher than that of the control group (12.82 *vs.*9.10, *p* < 0.05 or 12.33 *vs.* 8.20, *p* < 0.05, Table 3), whether unadjusted or adjusted by creatinine. These results could clearly distinguish the exposed group from the unexposed group. But the difference of the geometric mean value of urinary perchlorate between the two groups was not significant, whether adjusted or unadjusted by creatinine (data not shown, *p* = 1.0). Furthermore, the geometric mean and median value of urinary perchlorate in exposed workers at the end of shift was significantly higher when compared with that of before the shift (6.99 *vs*. 2.23 and 10.51 *vs*. 8.89, *p* < 0.05, Table 4), showing that the level of urinary perchlorate was markedly increased across the shift. 

**Table 3:** Pre-shift mean and median values of urinary perchlorate concentrations of each group (unadjusted and adjusted by creatinine).

Urinary perchlorate ^△^	Exposed (n = 51)	Control (n = 41)	T-Values
Mean ± SD (µg/L)	12.82 ± 0.37 *	9.10 ± 0.48	41.986
Median (range) (µg/L)	8.89 (0.92–24.71)	5.56 (0.37–20.55)	NA
Mean ± SD (μg/g Cr)	12.33 ± 0.68 *	8.20 ± 0.70	28.58
Median (range) (μg/g Cr)	8.51 (0.88–23.76)	4.96 (0.33–18.47)	NA

Notes: ^△^ The urinary perchlorate determined were measured before the shift. NA, not applicable. * *p* < 0.05, *vs.* controls.

**Table 4:** The comparison of perchlorateconcentration inthe urine for occupational workers at the differentwork shifts ^a^ (µg /L).

Sampling time	95% CI	Median	Max	Min
Before shift (n = 51)	1.64–23.98	8.89	24.71	0.92
End of the shift (n = 21)	5.69–35.5	10.51	35.97	5.09
*p* Values	NA	NA	NA	NA

Notes: ^a^ Urinary perchlorate is not normally distributed, and presented by median, max, min and 95% CI (confidence interval). NA not applicable.

### 3.4. Serum Biochemistry and Hematology Profiles

As presented in Table 5, the results of serum chemistry and hematology profiles were within normal limits throughout the study in all participants. Interestingly, compared with the control group, the significantly higher mean level of blood urea nitrogen (BUN) was observed within the exposed group (*p* = 0.001), while the BUN values of the two groups were within the normal reference range, which make no sense from a clinical viewpoint.

**Table 5:** Mean values of serum chemistry and hematology parameters of workers from the exposed group and control group (mean ± SD).

Parameters	Group (Mean ± SD)		*p-*Values
	Exposed (n = 51)	Control (n = 41)	
WBC (×10^9^/L)	5.59 ± 1.27	5.87 ± 1.16	0.287
RBC (×10^12^/L)	4.44 ± 0.40	4.53 ± 0.42	0.297
Hb (g/L)	130.08 ± 15.67	132.80 ± 14.42	0.392
PLT (×10^9^/L)	224.65 ± 141.38	226.54 ± 91.06	0.941
GPT (U/L)	19.06 ± 7.68	21.56 ± 10.57	0.193
BUN (mmol/L)	5.27 ± 1.46	4.30 ± 1.29	0.001 **
Cr (μmol/L)	98.87 ± 24.93	92.30 ± 24.09	0.206
ALB (g/Dl)	44.74 ± 2.60	44.01 ± 2.72	0.196

Notes: *p-*values obtained from separate logistic regression models for each variable; ** *p* < 0.01, *vs.* controls.

### 3.5. Thyroid Function Status

Results of thyroid function status are presented in Table 6. The difference of the TPO antibody positive rate in the two groups was not significant (*p* = 0.70), indicating that the two groups were comparable. The comparison of FT_3_, FT_4_, log (TSH) and Tg levels showed no significant difference between the two groups (*p* = 0.953, *p* = 0.120, *p* = 0.35 and *p* = 0.401, respectively). The TSH (µU/mL) (Median (range)) of exposed group and control group was 3.75 (0.75 to 13.62) vs. 2.68 (0.33 to 5.78), respectively. Further, there is no significant difference in their distributions (*p* = 0.35).

**Table 6:** Statistical description of the thyroid function indicators of workers from the exposed group and control group (Mean ± SD).

Parameters	Exposed (n = 51)	Control (n = 41)	*p-*Values
FT_3 _(fmol/L)	2.91 ± 1.41	2.92 ± 1.19	0.953
FT_4 _(fmol/L)	11.67 ± 4.46	13.14 ± 4.47	0.120
Log (TSH)(µU /mL) ^a^	0.369 ± 0.255	0.466 ± 0.311	0.35
Tg (ng/mL）	6.27 ± 2.82	5.89 ± 0.90	0.401
TPO antibody positive rate (%)	3/51(5.9)	4/41(9.8)	0.70

Note: ^a^ TSH is not normally distributed, and subject to the logarithmic transformation.

### 3.6. Urinary Iodine Status

Table 7 shows that the urine iodine concentration of the two group of workers did not differ significantly (*p* = 0.576 for un-adjusted by creatinine, and *p* = 0.742 for adjusted by creatinine). In addition, whether adjusted by creatinine or not, the median values of urinary iodine of the exposed group were slightly lower than those of the control group, but the difference was not statistically significant.Based on the World Health Organization standards, the two groups are iodine sufficient. The ranges of urine iodine of the exposed group and control group were 77.97–327.08 (µg/g Cr) or 79.0–273.0 (µg/L) and 100.76–595.2 (µg/g Cr) or 85.4–291.8 (µg/L), respectively. 

**Table 7:** Mean and median values of urinary iodine (UI) concentrations of each group (unadjusted and adjusted by creatinine).

Parameters	Exposed (N = 51)	Control (N = 41)	*p-*Values
UI (µg /L) Mean ± SD	172.47 ± 105.22	184.29 ± 105.22	0.576
UI (μg/g Cr ) Mean ± SD	179.96 ± 85.42	186.47 ± 99.51	0.742
UI (µg /L) Median	155.4 ^a^	161.0 ^a^	NA
UI (μg/g Cr) Median	154.6	159.1	NA
Range (µg/L)	79.0–273.0	85.4–291.8	NA
Range (µg/g Cr)	77.97–327.08	100.76–595.2	NA

Note: ^a^ median urinary iodine levels > 100 μg/L, indicating of sufficient iodine intake for a population according to the WHO criteria. NA, not applicable.

### 3.7. Pulmonary Function Profiles

As evidenced in Table 8, no significant difference in means of pulmonary function indicators such as FVC%, FEV1% and FEV1/FVC% were observed in the exposed group, when compared to the control group (*p* > 0.05).

**Table 8:** The results of pulmonary functiontest in two groups in Yicheng Survey (Mean±SD).

Group	Numbers	FVC (%)	FEV1 (%)	FEV1/FVC (%)
Exposed	51	99.66 ± 12.81 *	98.53 ± 16.71 *	98.92 ± 11.30 *
Control	41	95.50 ± 17.59	94.71 ± 18.26	99.17 ± 14.38
*p*-values	NA	0.192	0.298	0.346

Notes: FVC, forced vital capacity; FEV1, forced expiratory volume in one second; FEV1/FVC, forced expiratory volume in one second to forced vital capacity ratio; NA, not applicable. * *p* *>* 0.05, *vs.* controls.

## 4. Discussion

To our knowledge, this is the first report of examining a biomarker of perchlorate exposure for an association with an effect on thyroid homeostasis of Chinese workers. The results of serum chemistry and hematology profiles were within normal limits throughout the study in all participants, which is consistent with the previous findings [14]. The primary thyroidal parameters (FT_3_, FT_4_, Log TSH, Tg, and TPO) showed no significant differences between the exposed workers and the controls. 

Iodide is required to synthesize hormones critical for fetal and neonatal development. The human thyroid gland contains a huge reserve store of hormone within the intrafollicular thyroglobulin. If iodine intake is sufficient, even a reduction in thyroidal iodide concentration to one-third of its former level may still leave these individuals sufficient iodide to produce thyroid hormone at a normal rate. As shown in Table 7, the iodine nutritional status of the two groups was sufficient. Further, given that perchlorate has a half-life of about 8–14 h, the plant workers, many of whom worked 12 h shifts, might recover from the inhibitory effects of exposure during off-shift periods.

In this study, no significant difference was observed between the two groups in the main indicators of thyroid function such as FT_3_, FT_4_ and TSH. Historically, the mode of action analysis suggests that alteration of hormones (T_4_, T_3_ and TSH) would be the first observed biological effect of perchlorate exposure [23]. TT_4_ was measured in addition to thyroid parameters shown in Table 4. The mean TT_4_ level for the exposed workers (65.6 ± 16.7 ng/mL) was significantly (*p* = 0.034) lower than the mean TT4 level for the controls (81.9 ± 46.2 ng/mL). Bruce *et al.* [24] demonstrated that depressed TT4 levels are due to increased nitrate and thiocyanate levels and not to increased perchlorate levels. Neither serum nor urinary levels of nitrate or thiocyanate were measured in this study. A second explanatory hypothesis might be that lower TT_4_ levels reflect lower thyroid binding globulin levels, but that also was not measured. Because of the lack of determination of TBG, the exact cause of decrease in total T4 remain unclear. Perchlorate is not known to be associated with binding globulin alterations, so the association observed in this study is not, in fact, in line with the known mode of action of perchlorate. Thus, further studies are needed to clarify these findings. 

Concurrent quantification of iodine and perchlorate levels in human urine can provide information on potential risks from perchlorate exposure. Urinary perchlorate is reasonably indicative of human exposure, because 70%–95% of a perchlorate dose is excreted unchanged in the urine with a half-life of 8–14 h [8,25], not only urine collection is less invasive than blood collection, but also urinary perchlorate levels tend to be much higher than serum levels due to efficient renal clearance of perchlorate. Urinary perchlorate levels are elevated in the urine because the kidney reabsorbs water from the urine and thus concentrates the urinary solutes. Measuring perchlorate in human urine can assess the combined exposure from all sources [26]. By comparing the concentration of urinary perchlorate at the end of shift and before the shift could reflect the exposure intensity during the work shift. The geometric mean concentration of urinary perchlorate in the exposure group was markedly higher than that of the control group, which demonstrated a direct association between urinary levels of perchlorate and AP exposure dose in workplace (Table 4).

Perchlorate is not metabolized in the human body, and is excreted from the urine, with a biological half-life of approximately 8 hours. So the timing of perchlorate exposure is important for individual exposure assessment. The pre-shift urine samples were obtained in the early morning after a day of consecutive work and adequate rest in the evening. The urine perchlorate of the control group was detected, indicating the presence of background exposure. The perchlorate content in the waste water discharged from the plant was also detected, confirming the source of exposure (data not shown). The concentration of urinary perchlorate in the exposure group was markedly higher than that of the control group, which showed that urinary perchlorate is a specific biomarker to distinguish exposed from unexposed individuals.

We found no evidence of an effect on urinary iodine from environmental perchlorate dust exposure in workplace. Iodine nutrition status is crucial to the comparative evaluation of the thyroid function of the two groups. However, iodine levels in spot urine can vary significantly from sample to sample, due to variation in iodine intake throughout the day and from day to day. In the region studied, the main source of iodine intake is iodized salt ingestion. In addition, urinary iodine concentrations can be affected by fluid status, Therefore, creatinine adjustments were used to account for differences in fluid volume status. The median urinary iodine levels of the two groups of workers were close to each other; additionally, the difference was not statistically significant, indicating that the iodine nutrition status of the two groups was almost identical, and had strong comparability.

A previous study has shown that creatinine adjustment for estimation of 24 h excretion from spot samples was not effective for iodine [27]. However, another study revealed that single-spot urine samples could reflect the participant’s exposure level, providing support for the use of a single sample as an exposure measure in epidemiological studies [28]. Owing to the slight decrease of median of urinary iodine in the exposed group comparing with that of the control group, our research showed that spot urinary iodine might be used as an indicator of the iodine nutrition status of occupational workers.

The high iodine levels found in the urine of some people (maximum concentration: 595.2 g/g Cr) indicated that effective measures of iodized salt were widely implemented in China. The median urinary iodine concentration in a population provides a good yardstick for its current iodine nutrition. The WHO (2007) has identified median urinary iodine levels ≥ 100 μg/L as an indicator of sufficient iodine intake for a population, while median urinary iodine levels > 300 μg/L indicate excessive iodine intake [29]. In this study elevated concentrations of iodine (>300 μg/L) were found in five of the urinary samples from the total participants; the concentrations ranged from 320.4 to 480.2 μg/L. This elevated concentrations of urinary iodine indicated that some individuals may have ingested large amounts of iodine from their diet. Furthermore, such high iodide intake may effectively compete with perchlorate binding sites on the NIS, which generally alleviates the effects of perchlorate on thyroid homeostasis.

There have been reported that high dose of ammonium perchlorate dust exposure could lead to pulmonary fibrosis in Chinese workers [30]. Our results were inconsistent with the previous study, and possible reason lies in the significant decreased dust concentration due to the proper use of protective equipment and the improvement of the production process.

The present study has the general limitations of a cross-sectional analysis. Therefore, the relationship between perchlorate exposure and thyroid function was examined with attention to the potential influences of chance, bias, or confounding. Further, thiocyanate exposure was not actually measured, which was the major source of sodium iodide transporter inhibitors. A larger sample size might help to average such potential kinetic differences. Limitations also include a lack of measurements of specific chemical components (TBG) that may have been responsible for the level of TT_4_ in serum samples, as well as the limitation of a 2-day monitoring of ammonium perchlorate concentration in the dust in the workplace.

## 5. Conclusions

The findings of the present study highlight the importance of iodine in thyroid homeostasis in occupationally AP exposed workers, as previously reported in animal experiments and in human studies [31,32]. Furthermore, the significant increase of median value of urinary perchlorate at the end of the shift was observed when compared with that of before the shift, which suggested the perchlorate in the urine can be used as the biomarker indicating exposure intensity. It has been confirmed that serum free T_4_ decrease is a key effect of AP absorption, based on a mode-of-action analysis and the evidence provided by studies on rodents [31]. However, in this study, free T_4_ did not decrease, suggesting that NIS inhibition was insufficient to initiate the mode of action above mentioned. To our knowledge this is the first study focused on the indicators of AP exposure of occupationally exposed workers in China, which examines for an effect on the thyroid function of Chinese workers of occupational AP exposure, and the interaction with iodine. However, more studies and more extensive investigations are needed to elucidate the issue, since human studies are scarce. Further research to determine more deeply the correlation between ammonium perchlorate exposure and thyroid function would be helpful in defining any possible public health risk.

## References

[1] Parker D.R. (2009). Perchlorate in the environment: The emerging emphasis on natural occurrence. Environ. Chem..

[2] Dasgupta P.K., Martinelango P.K., Jackson W.A., Anderson T.A., Tian K., Tock R.W., Rajagopalan S. (2005). The origin of naturally occurring perchlorate: The role of atmospheric processes. Environ. Sci. Technol..

[3] Kirk A.B., Martinelango P.K., Tian K., Dutta A., Smith E.E., Dasgupta P.K. (2005). Perchlorate and iodide in dairy and breast milk. Environ. Sci. Technol..

[4] Dasgupta P.K., Dyke J.V., Kirk A.B., Jackson W.A. (2006). Perchlorate in the United States: Analysis of relative source contributions to the food chain. Environ. Sci. Technol..

[5] Murray C.W., Egan S.K., Kim H., Beru N., Bolger P. (2008). MUS Food and Drug Administration’s total diet study: Dietary intake of perchlorate and iodine. J. Exp. Sci. Environ. Epidemiol..

[6] Gibbs J.P., Ahmad R., Crump K.S., Houck D.P., Leveille T.S., Findley J.E., Francis M. (1998). Evaluation of a population with occupational exposure to airborne ammonium perchlorate for possible acute or chronic effects on thyroid function. J. Occup. Environ. Med..

[7] Charnley G. (2008). Perchlorate: Overview of risks and regulation. Food Chem. Toxicol..

[8] Urbansky E.T., Schock M.R. (1999). Issues in managing the risks associated with perchlorate in drinking water. J. Environ. Manage..

[9] Wolff J. (1998). Perchlorate and the thyroid gland. Pharmacol. Rev..

[10] Driguezh A.F., Davidson H.G., Villadiego M.S., Fernandez A.M., Lacave I.M., Sanz J.F. (1991). Induction of thyroid proliferative changes in rats treated with antithyroid compound. Anat. Histol. Embryol..

[11] Zhou X., Wu F.H., Zhang R., Pan Z.M., Peng K.L. (2011). Effects of ammonium perchlorate on iodine uptake and antioxidant capacity of thyroid. J. Environ. Occup. Med..

[12] Chen Y., Sible J.C., McNabb F.M. (2008). Effects of maternal exposure to ammonium perchlorate on thyroid function and the expression of thyroid-responsive genes in Japanese quail embryos. Gen. Comp. Endocrinol..

[13] Tonacchera M., Pinchera A., Dimida A., Ferrarini E., Agretti P., Vitti P., Santini F., Crump K., Gibbs J. (2004). Relative potencies and additivity of perchlorate, thiocyanate, nitrate, and iodide on the inhibition of radioactive iodide uptake by the human sodium iodide symporter. Thyroid.

[14] Lawrence J.E., Lamm S.H., Pino S., Richman K., Braveman L.E. (2000). The effect of short-term low-dose perchlorate on various aspects of thyroid function. Thyroid.

[15] Scinicariello F., Murray H.E., Smith L., Wilbur S., Fowler B.A. (2005). Genetic factors that might lead to different responses in individuals exposed to perchlorate. Environ. Health Persp..

[16] Trumbo P.R. (2010). Perchlorate consumption, iodide status, and thyroid function. Nutr. Rev..

[17] Greer M.A., Goodman G., Pleus R.C., Greer S.E. (2002). Health effects assessment for environmental perchlorate contamination: The dose response for inhibition of thyroidal radioiodine uptake in humans. Environ. Health Persp..

[18] Braverman L.E., He X.M., Pino S., Cross M., Magnani B., Lamm S.H., Kruse M.B., Engel A., Crump K.S., Gibbs J.P. (2005). The effect of perchlorate, thiocyanate and nitrate on thyroid function in workers exposed to perchlorate long-term. J. Clin. Endocrinol. Metab..

[19] Pino S., Fang S.L., Braverman L.E. (1996). Ammonium persulfate: A safe alternative oxidizing reagent for measuring urinary iodine. Clin. Chem..

[20] Bartels H., Bohmer M., Heierli C. (1972). Serum creatinine determination without protein precipitation. Clinica Chimica Acta.

[21] Yang L., Liu Z., Chen W., Chen J., Li J., Yi G., Yang J., Mei Y., Qi C., Peng K., Liu J., Ye B. (2007). Total Dust Concentration. Method for Determination of Dust in the Air of Workplace.

[22] Valentin-Blasini L., Mauldin J.P., Maple D., Blount B.C. (2005). Analysis of perchlorate in human urine using ion chromatography and electrospray tandem mass spectrometry. Anal. Chem..

[23] (2008). Toxicological Profile for Perchlorate.

[24] Bruce G.M., Corey L.M., Mandel J.H., Pleus R.C. (2013). Urinary nitrate, thiocyanate, and perchlorate and serum thyroid endpoints based on NHANES 2001 and 2002. J. Occup. Environ. Med..

[25] Lamm S.H., Braverman L.E., Li F.X., Richman K., Pino S., Howearth G. (1999). Thyroid health status of ammonium perchlorate workers: A cross-sectional occupational health study. J. Occup. Environ. Med..

[26] Blount B.C., Valentin-Blasini L. (2007). Using biomonitoring as a method for assessing exposure to perchlorate. Thyroid.

[27] Ohira S.I., Kirk A.B., Dyke J.V., Dasgupta P. (2008). Creatinine adjustment of spot urine samples and 24 h excretion of iodine, selenium, perchlorate, and thiocyanate. Environ. Sci. Technol..

[28] Mervish N., Blount B., Valentin-Blasini L., Brenner B., Galvez M.P., Wolff M.S., Teitelbaum S.L. (2011). Temporal variability in urinary concentrations of perchlorate, nitrate, thiocyanate and iodide among children. J. Expo. Sci. Environ. Epidemiol..

[29] (2007). Assessment of Iodine Deficiency Disorders and Monitoring Their Elimination: A Guide for Programme Managers.

[30] Peng K.L., Lu X.M., Zhao P.L., Zhao S.L., Lin M.F., Wang Z.L., Wei Z.H., Guo H.X. (2009). Effect of ammonium perchlorate dust on the health status of exposed workers. Ind. Health Occup. Dis..

[31] Wu F.H., Zhou X., Zhang R., Pan M.Z., Peng K.L. (2012). The effects of ammonium perchlorate on thyroid homeostasis and thyroid-specific gene expression in rat. Environ. Toxicol..

[32] Mendez W., Eftim S.E. (2012). Biomarkers of perchlorate exposure are correlated with circulating thyroid hormone levels in the 2007–2008 NHANES. Environ. Res..

